# Sensor-guided gap balance versus manual gap balance in primary total knee arthroplasty: a meta-analysis

**DOI:** 10.1186/s13018-022-03129-x

**Published:** 2022-04-19

**Authors:** Changjiao Sun, Zhe Zhao, Woo Guan Lee, Qi Ma, Xiaofei Zhang, Jianjin Zhu, Xu Cai

**Affiliations:** 1 Department of Orthopedic, Beijing Tsinghua Changgung Hospital, School of Clinical Medicine, Tsinghua University, No. 168 Litang Road, Dongxiaokou Town, Changping District, Beijing, 102218 China; 2 FRCS (Edinburgh), Kuching Specialist Hospital, Tabuan Stutong Commercial Centre, 93350 Kuching, Sarawak Malaysia

**Keywords:** Total knee arthroplasty, Sensor, Sensor-guided, Manual, Gap balance

## Abstract

**Background:**

Despite Vast improvements in technology and surgical technique in total knee arthroplasty (TKA), approximately 15–25% TKAs, have suboptimal subjective clinical outcomes. Our study sought to evaluate if sensor-guided balancing improves postoperative clinical outcomes compared to a conventional gap balancing technique.

**Methods:**

We searched Web of Science, Embase, PubMed, Cochrane Controlled Trials Register, Cochrane Library, Highwire, CBM, CNKI, VIP, and Wanfang database in March 2022 to identify studies involving sensor-guided balancing versus conventional gap balancing technique in TKA. Finally, we identified 2147 knees assessed in nine studies.

**Results:**

Compared with manual gap balancing, Sensor-guided gap balancing resulted in less rate of Manipulation under anesthesia (MUA) (*P* = 0.02), however more rate of intraoperative additional procedures (*P* = 0.0003). There were no significant differences in terms of KSS (*P* = 0.21), KSS Function score (*P* = 0.36), OKS (*P* = 0.61), KOOS (*P* = 0.78), operative time (*P* = 0.17), Mechanical axis (*P* = 0.69) and rate of reoperation between two groups.

**Conclusion:**

Compared with conventional manual gap balancing techniques, sensors have more balancing procedures being performed. However, it did result in a reduction in the rate of MUA. More extensive, high-quality RCTs are required to verify our findings further.

## Introduction

Total knee arthroplasty (TKA) has proven to be a successful operation in significantly reducing osteoarthritic knee pain and is more cost-effective than prolongation with nonsurgical treatments [1]. However, 15–25% of patients undergoing TKA report dissatisfaction after their procedure [2], and this dissatisfaction occasionally be due to soft tissue imbalance [2, 3]. Proper soft tissue balancing is the most critical contributor to improved outcomes after TKA [3, 4]. It was estimated that soft tissue imbalance causes up to 35% of early TKA revisions., manifesting as stiffness, instability, or tibiofemoral incongruency [5–8]. Although soft tissue balancing is essential, it is often determined by the surgeon's subjective "feel" of the local ligamentous tension. It typically depends on operative experience [9, 10]. To address this problem and to make ligament balancing less operator dependent, newer technologies such as patient-specific instrumentation [11, 12], computer-assisted surgery [13], and intraoperative pressure sensors [14] have been developed over the past decades. Intraoperative pressure sensors were introduced in TKA surgery to quantify compartmental pressures through a range of motion and determine tibiofemoral congruence. Several studies have shown improved early results with sensors [15–18], whereas others have failed to demonstrate a clinical benefit compared with the conventional gap balancing technique [19–23]. However, no meta-analysis studies have compared sensor-guided gap balancing with traditional manual knee balancing. Hence, this meta-analysis aims to determine whether sensor-guided gap balancing confers a clinical benefit compared with conventional manual gap balancing, as determined based on improved postoperative clinical outcomes.

## Methods

The study was conducted by the guidelines of the Preferred Reporting Items for Systematic reviews and Meta-Analyses (PRISMA) statement [24]. The protocol for this study was registered at PROSPERO (the International Prospective Register of Systematic Reviews), and the registration number was CRD 42021262271.

### Search strategy

We searched Web of Science, Embase, PubMed, Cochrane Controlled Trials Register, Cochrane Library, Highwire, CBM, CNKI, VIP, and Wanfang database in March 2022 to identify studies involving sensor-guided balancing versus conventional gap balancing technique in TKA. The keywords used were "total knee arthroplasty," "total knee replacement," "gap balancing," "sensor," sensor-guided," "manual in conjunction with Boolean operators, "AND" or "OR." Review Manager Software was used to perform the meta-analysis.

### Inclusion criteria

We identified and included all randomized controlled trials (RCTs) and non-randomized controlled trials (non-RCTs) comparing sensor-guided gap balancing (SB) and manual gap balancing (MB) in primary TKA in the search strategy. Studies were included for further assessment if they satisfied the following criteria: (1) The TKA procedure was performed for the first time. (2) sensor-guided gap balancing was involved. (3) The comparator was manual gap balancing in the comparative study. (4) At least one of the following indexes was reported: Knee society score (KSS); Knee society function score (KSFS); Oxford knee assessment (OKS); knee injury and osteoarthritis score (KOSS); Operative time; Mechanical axis; Intraoperative additional procedures (additional soft tissue releases or bone recuts); Manipulation under anesthesia (MUA); Reoperation. We also excluded: (1) studies that revision of TKA was performed. (2) unclear or incomplete sample data were available.

### Data extraction process

All RCTs and n RCTs comparing SB and MB in primary TKA were identified and included in the search strategy. Two independent investigators screened each of the studies for inclusion in the meta-analysis, and they independently extracted the available data from each study. Data were extracted based on the following: (1) research features (i.e., authors, type of study, year of publication), (2) population information (i.e., gender, body mass index [BMI], age), (3) outcome. We will contact the authors by email or other means to obtain more data if the necessary results are omitted.

### Assessment of studies

To assess the methodological quality, we evaluated the non-randomized studies using the nine-star Newcastle–Ottawa Scale (NOS), a validated tool suitable for evaluating the quality of non-randomized studies^21^. The methodological quality and basis of the RCTs were assessed according to the Cochrane Handbook for Systematic Reviews of Interventions. Two independent investigators assessed the quality of each study, and a third investigator resolved any discrepancies.

### Statistical analysis

We used the *I*^2^ and Q test to evaluate the heterogeneity between studies. *P* ≤ 0.1 or *I*^2^ value > 50% suggested high heterogeneity; thus, we used the randomized-effects model. Otherwise, we used the fixed-effects model^20^. In each study, we used the odds ratio (OR) and relevant 95% confidence interval (CI) to measure dichotomous variables such as rates of intraoperative additional procedures, MU, and reoperation. Reported OR was supposed to approximate RR (relative risk) based on Cornfield's rare disease outcome assumption because the outcome is rare^23^. We used the mean difference (MD) or standard MD to assess continuous outcomes such as KSS, KSS function, OKS, KOOS, ROM, Operative time, and mechanical axis with a 95% confidence interval (CI). We used some statistical algorithms to estimate the standard deviation for those studies that provided only continuous variables for means and range^24^. We considered the results a statistically significant difference if P values were less than 0.05. Sensitivity analysis was used to assess the stability of the results (if necessary). We performed all statistical analyses with Review Manager (version 5.4 for MAC, the Cochrane Collaboration, Copenhagen).

## Results

### Search results

**Fig. 1:**
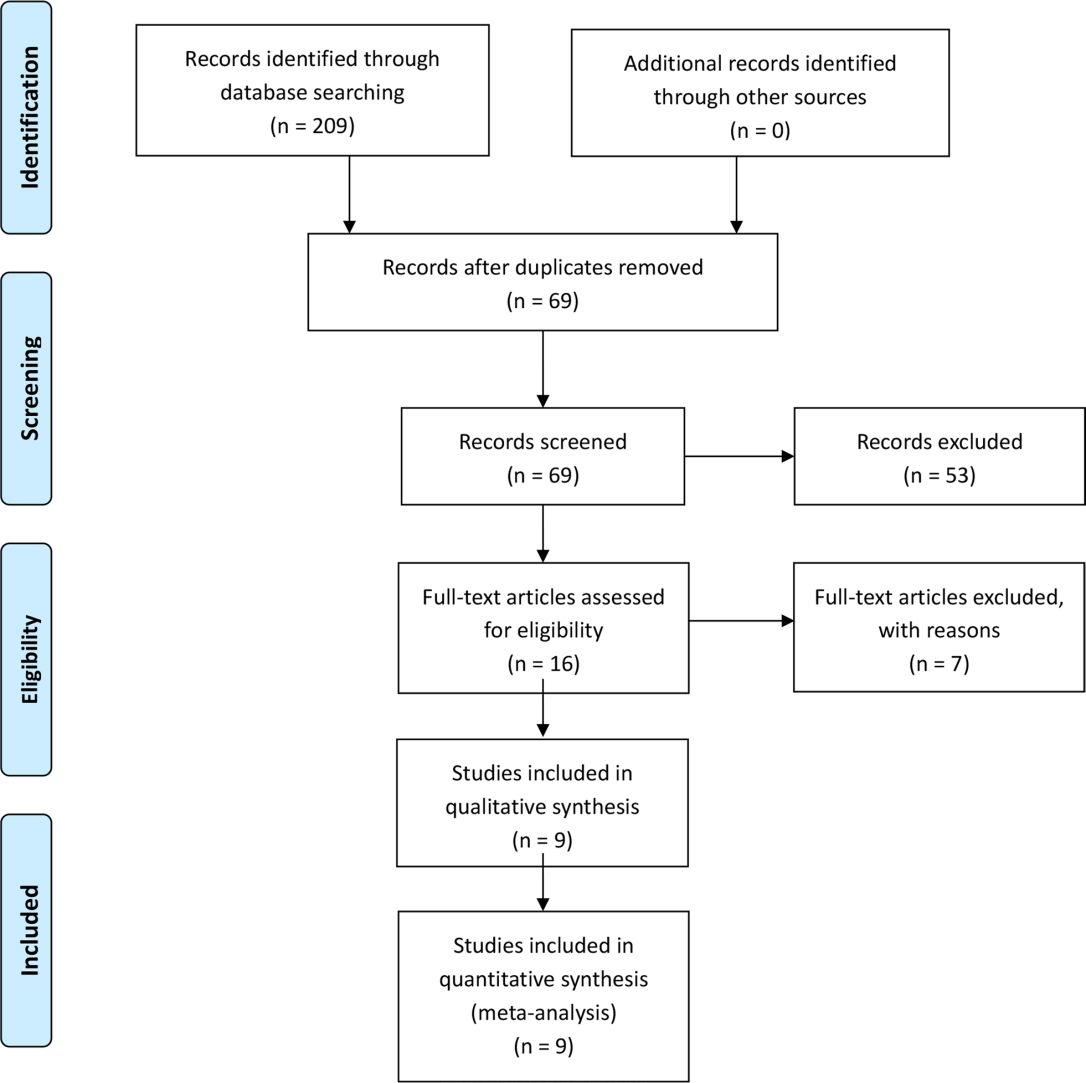
The literature search and selection process

The literature search and selection process are shown in Fig. 1. Finally, nine publications from 2016 to 2022 were included in our meta-analysis. The detailed literature screening process is shown as the PRISMA flow diagram in Fig. 1. 209 relevant citations were identified from the databases according to the literature search strategy described earlier. After deleting 140 duplicates, we obtained 69 articles. Upon review of titles and abstracts of the 69 remaining articles, 53 irrelevant clinical studies were excluded. By reading the 16 full-text articles, we excluded another seven articles for the following reasons: systematic reviews, no compare groups, cadaver researches, and no useful outcome data. The remaining nine articles were deemed appropriate. Finally, we identified 2147 patients (2147 knees) assessed in (4 RCTs [16, 18, 22, 23] and 5 non-RCTs [15, 17, 19–21]). All the articles were published in English and Chinese.

### Study characteristics and quality

We presented detailed baseline characteristics and general intervention information in Tables 1, 2 and 3. All the articles were published in English and Chinese between 2016 and March 2022.

**Table 1:** The detailed baseline characteristics information

Author/year	Sensor guided/manual					Outcome
	Patients	Knees	Mean age(years)	Female gender(%)	BMI	
Chow (2017)	57/57	57/57	67.6/66.1	52.6/59.65	29.5/29.4	9
Cochetti (2020)	50/50	50/50	67.7/67.3	2/6	34.4/34.7	1, 3, 5, 6, 8,9
Elmalah (2016)	10/12	10/12	64/66	NA	32/34	8
Geller (2017)	252/690	252/690	69/67	79/75	31/32	5, 6, 9
Keggi (2021)						
Livemore (2020)	74/194	74/194	69/65	54.1/57	31/29	4, 5, 9, 10
MacDessi (2020)	215/194	215/194	67.8/66.8	67.1/57	29.8/30	4, 8, 9, 10
Song (2018)	50/50	50/50	72.1/73	90/80	26/26.3	1, 2, 5, 7, 8
Wood (2020)	76/76	76/76	67.1/66.7	52.6/56.6	32.3/33.8	1, 3, 5, 8, 9,10
Xia (2019)	20/20	20/20	64.3/64.2	35/70	26.5/25.9	1, 2, 6, 7

BMI, Body Mass Index; TKA, total knee arthroplasty; KSS, Knee Society Score; KSFS, Knee Society Function Score; OKS, Oxford Knee Assessment; KOSS, Knee Injury and Osteoarthritis Score; MUA, manipulation under anesthesia

The detailed baseline characteristics information including the number of patients, TKAs, age, gender, BMI, outcome of two groups

1, KSS; 2, KSS function; 3, OKS; 4, KOOS; 5, ROM; 6, Operative time;7, Mechanical axis;8, Intraoperative additional procedures; 9, Rate of MUA;10, Rate of reoperation

**Table 2:** The detailed information of surgery

Author/year	Sensor	Prothesis	Diagnosis	Patellar resurfacing
Chow (2017)	VERASENSE^1^	﻿CR, JOURNEY II (﻿Smith & Nephew)	﻿ OA	Yes
Cochetti (2020)	﻿VERASENSE^1^	﻿PS, Persona ﻿(Zimmer-Biomet)	OA	NA
Elmalah (2016)	VERASENSE^1^	CR, ﻿Triathlon(Stryker Orthopedics)	OA, RA, POA	Yes
Geller (2017)	VERASENSE^1^	NA	NA	NA
Livemore (2020)	VERASENSE^1^	CR, ﻿Vanguard ﻿(Zimmer-Biomet)	OA	Yes
MacDessi (2020)	VERASENSE^1^	PS, ﻿Legion (﻿Smith & Nephew)	OA	Yes
Song (2018)	VERASENSE^1^	PS, NexGen (Zimmer)	OA	Yes
Wood (2020)	VERASENSE^1^	CR, ﻿Triathlon(Stryker Orthopedics)	OA	NA
Xia (2019)	REP 6032^2^	PS, XN(Beijing Chunli)	OA	No

OA, osteoarthritis; RA, rheumatoid arthritis; POA, post-traumatic arthritis; PS, posterior-stabilized; CR, cruciate-retaining

The detailed information of surgery including sensor, prothesis, diagnosis and patellar resurfacing of two groups

^1^Orthosensor, Dania Beach, Florida, USA

^2^Yubo Intelligent Technology, Hangzhou, China

**Table 3:** Risk-of-bias assessment for the studies included in the meta-analysis (NOS)

(Non-RCT) study = 5	Selection				Comparability	Outcome/exposure			Score
	Item 1	Item 2	Item 3	Item 4	Item 5	Item 6	Item 7	Item 8	
Chow (2012)	*	*	*	*	*	*			6
Cochetti (2020)	*	*	*	*	*	*		*	7
Geller (2017)	*	*	*	*	*	*		*	7
Livemore (2020)	*	*	*	*	*	*		*	7
MacDessi (2020)	*	*	*	*	*	*		*	7

The methodological quality of the involved studies ranged from 6 to 7

* means 1 point of score

### Risk of bias assessment

The methodological quality of the involved studies ranged from six to seven (Table 4). The risk of bias summary and risk of bias graph for RCTs are shown in Table 4. As a result, the overall quality of the included studies was considered adequate.

**Table 4:** Methodological assessment according to six domains of potential biases (Cochrane risk of bias tool)

RCT study = 4	Random sequence generation	Allocation concealment	Blinding of participants and personnel	Blinding of outcome assessment	Incomplete outcome data	Selective reporting	Other bias
Elmallah (2016)	Low	Low	High	Low	Low	Low	Unclear
Song (2018)	Low	Low	High	Low	Low	Low	Unclear
Wood (2020)	Low	Low	High	Low	Low	Low	Unclear
Xia (2019)	Low	Low	High	Low	Low	Low	Unclear

### KSS

**Fig. 2:**
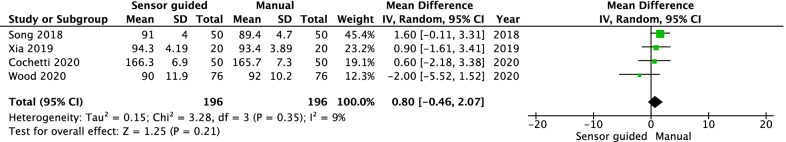
The pooled data showed that the KSS was not significantly different between the two groups (MD = 0.8 95% CI [− 0.46, 2.07], *P* = 0.21)

Four studies reported KSS; The pooled data showed that the KSS was not significantly different between the two groups (MD = 0.8 95% CI [− 0.46, 2.07], *P* = 0.21; Fig. 2).

### KSS function score

**Fig. 3:**
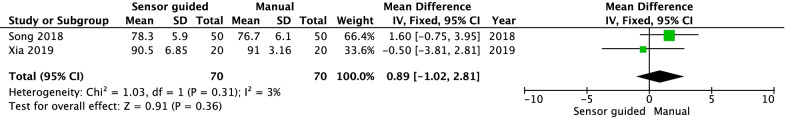
The forest plot revealed that both groups experienced similar KSS function scores (MD = 0.89, 95% CI [− 1.02, 2.81], *P* = 0.36)

Two studies reported the KSS function score. The forest plot revealed that both groups experienced similar KSS function scores (MD = 0.89, 95% CI [− 1.02, 2.81], *P* = 0.36; Fig. 3).

### OKS score

**Fig. 4:**
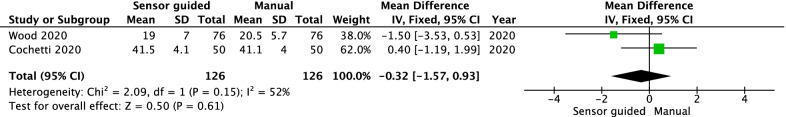
The forest plot revealed that both groups experienced similar OKS scores (MD = − 0.32, 95% CI [− 1.57, 0.93], *P* = 0.61)

Two studies reported the OKS. The forest plot revealed that both groups experienced similar OKS scores (MD = − 0.32, 95% CI [− 1.57, 0.93], *P* = 0.61; Fig. 4).

### KOOS

**Fig. 5:**
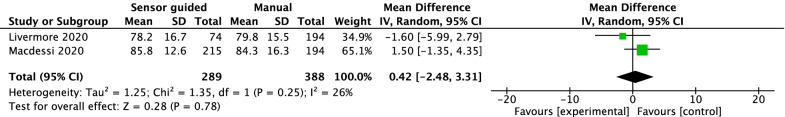
The forest plot revealed that both groups experienced similar KOOS scores (MD = 0.42, 95% CI [− 2.48, 3.31], *P* = 0.78)

Two studies reported the KOOS. The forest plot revealed that both groups experienced similar KOOS scores (MD = 0.42, 95% CI [− 2.48, 3.31], *P* = 0.78; Fig. 5).

### Operative time

**Fig. 6:**
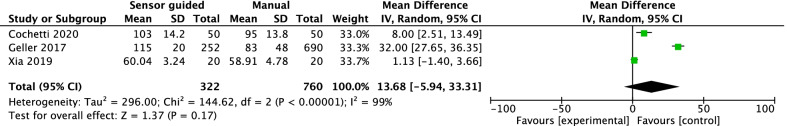
The forest plot revealed that both groups experienced a similar operative time (MD = 13.68, 95% CI [− 5.94, 33.31], *P* = 0.17)

Three studies reported the operative time. The forest plot revealed that both groups experienced a similar operative time (MD = 13.68, 95% CI [− 5.94, 33.31], *P* = 0.17; Fig. 6).

### Mechanical axis

**Fig. 7:**
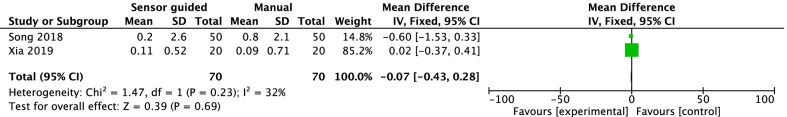
The forest plot revealed that the mechanical axis was not significantly different between the two groups (MD = − 0.07, 95% CI [− 0.43, 0.28], *P* = 0.69)

Two studies reported the mechanical axis. The forest plot revealed that the mechanical axis was not significantly different between the two groups (MD = − 0.07, 95% CI [− 0.43, 0.28], *P* = 0.69; Fig. 7).

### Intraoperative additional procedures

**Fig. 8:**
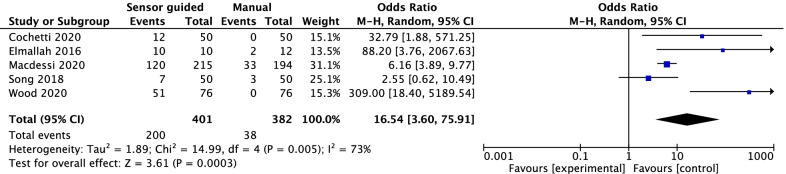
The forest plot revealed that the rate of intraoperative additional procedures was significantly more when the sensor was applied (OR = 16.54, 95% CI [3.6, 75.91], *P* = 0.0003)

Five studies reported intraoperative additional procedures. The forest plot revealed that the rate of intraoperative additional procedures was significantly more when the sensor was applied (OR = 16.54, 95% CI [3.6, 75.91], *P* = 0.0003; Fig. 8).

### Rate of MUA

**Fig. 9:**
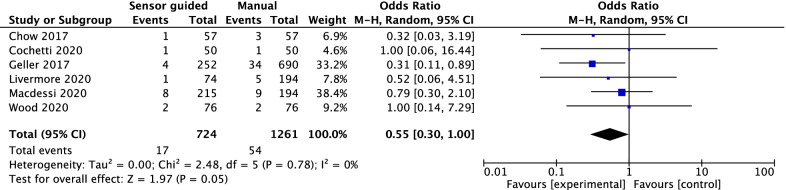
The forest plot revealed that the rate of MUA was significantly less when the sensor was applied (OR = 0.51, 95% CI [0.28, 0.91], *P* = 0.02)

Six studies reported the rate of MUA. The forest plot revealed that the rate of MUA was significantly less when the sensor was applied (OR = 0.51, 95% CI [0.28, 0.91], *P* = 0.02; Fig. 9).

### Rate of reoperation

**Fig. 10:**
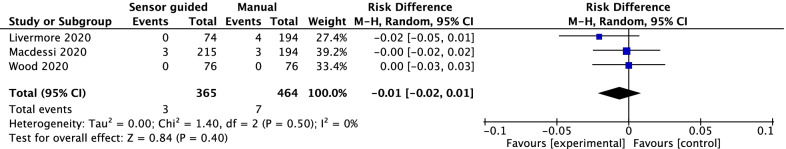
The forest plot revealed that both groups experienced a similar rate of reoperation (RD = − 0.01, 95% CI [− 0.02, 0.01], *P* = 0.4)

Three studies reported the rate of reoperation. The forest plot revealed that both groups experienced a similar rate of reoperation (RD = − 0.01, 95% CI [− 0.02, 0.01], *P* = 0.4; Fig. 10).

## Discussion

There is uncertainty and controversy about the influence of sensor-guided knee gap balancing and conventional gap balancing techniques on clinical outcomes following primary TKA. We sought to evaluate the body of evidence linking sensor-guided knee gap balancing versus conventional gap balancing technique following primary TKA, carrying out a comprehensive systematic review of RCTs and observational studies. To our knowledge, this is the first meta-analysis comparing the sensor-guided knee gap balancing and conventional manual gap balancing technique in primary TKA. This study showed that the use of an intraoperative sensing technology during primary TKA was not related to a statistically significant improvement in KSS, KSS Function score, OKS, KOOS, operative time, Mechanical axis, and rate of reoperation when comparing conventional soft tissue balancing. The most relevant finding was that compared with conventional manual gap balancing techniques, sensors have more balancing procedures (soft tissue releases or bone recuts) being performed. However, it did result in a reduction in the rate of MUA.

Continued advancements in technology and improvement in surgical techniques have made TKA surgery a very successful operation [25]. However, approximately one-third of early TKA revisions are related to unbalanced soft tissue presenting as stiffness, instability, or early component loosening [8, 26–31]. The ligament balancing "feeling" is affected by factors such as patient generalized laxity, degree of joint contracture, BMI, gender, the depth of anesthesia, surgical experience, and even the surgeon's favorite method of balancing(spacer blocks or ligament tensioners) [32, 33]. Optimizing soft-tissue balance in TKA is considered an essential surgical prerequisite to improve clinical outcomes. The laxities of the native knee are not uniform throughout the arc of motion [34], further suggesting that manual gap balancing techniques may not adequately address imbalances, thus emphasizing the need for a fresh look at soft tissue balancing [35]. Navigation provides data on a numerical gap, measured laterally and medially in flexion and extension before cuts are made, usually displayed in millimeters [36]. During a robotic-assisted procedure, the leg is physically manipulated to stress the collaterals in flexion and extension to assess the gap balancing. It is difficult to apply a valgus/varus stress test in flexion due to the inability to control hip rotation and assess it accurately [37]. Sensors offer surgeons different information from traditional navigation or robotic systems. The wireless, intraoperative sensor tibial insert consisting of two microelectronic sensors embedded into the tibial tray has been designed to provide intraoperative real-time feedback. After the tibial and femoral cuts are completed. The capsule is closed by a few stitches. The surgeon holds the leg in a neutral position, and the sensor tibial insert monitors the medial and lateral loading forces from full extension to full flexion. The sensor describes quantitative loads at major contact points in both compartments and peak center of load location during both TKA trials and final implant positioning [14]. The quantitative balance has been defined as a mediolateral intercompartmental loading difference of fewer than 15 pounds [14]. Using this technology, the surgeon receives real-time feedback of the loading in the knee and can adjust any imbalance with soft tissue corrections or additional bony resections [38, 39].

Several authors described the necessity of additional soft tissue release or bone recut to obtain the desired intra-articular loads [19]. In our study, there were more intraoperative additional procedures in the sensor groups, and thus theoretically should increase the patient clinical outcomes. But our meta-analysis didn't verify this hypothesis. We found no significant difference in KSS, KSS Function score, OKS, KOOS, operative time, mechanical axis, and rate of reoperation between two groups at short-term follow-up. However, our meta-analysis found that using the sensor did result in a reduction in MUA rate. Stiffness or arthrofibrosis is one of the most common complications associated with joint arthroplasty surgery [40]. Arthrofibrosis may occur due to several factors, including those on the part of the surgeon and patient [40, 41]. MUA is a therapeutic procedure to treat stiffness or arthrofibrosis [42]. The surgeon can loosen adhesions to reduce joint arthrofibrosis. ﻿In our meta-analysis, we chose to focus on MUA as one primary outcome of the utility of the sensor-enabled device. The emotional burden of arthrofibrosis or stiffness on the patient can be difficult, with many patients never obtaining an optimal clinical outcome. In addition, the cost burden cannot be overlooked. Based on 2014 Center's for Medicare & Medicaid Services (CMS) data, The average cost of MUA was close to $1200 per case [17]. Additionally, a majority (62%) of cases were within a 90-day postoperative window [19].

Since complications and readmissions before a 90-day threshold will result in a financial burden to the medical resource, sensor use demonstrates a potential to decrease the incidence of financial loss by mitigating early MUA.

Our findings should be considered with an understanding of the critical limitations of the data set. Firstly, we only included four randomized controlled trials; the other five studies were observational studies, which may have reduced the quality of the evidence for this meta-analysis. Although we have included all related studies thus far and tried to collect more data to make this meta-analysis and assess its effect, more prospective randomized trials investigating other clinical parameters are needed to confirm the results and conclusions. Secondly, there was an essential variability between the studies with respect to the different operating surgeons, different levels of TKA constraint (PCL substituting versus PCL retaining), the patient population, follow-up period, the cohorts evaluated, and the analyses performed. Thirdly, The follow-up period for these studies remains short, principally because this system is so new. Studies with longer follow-up and well-defined groups randomized to surgery performed with or without a sensor would provide valuable data for analysis. Furthermore, most of our included articles studied a specific type of sensor (Verasense) and these results may not be universally applicable to other sensor technologies on the market (e.g. Omnibot). The OMNIBot (Corin Ltd, Rayham, MA) has been shown to be a reliable tool for delivering different alignment philosophies as well as planning and achieving tibio-femoral coronal balancing [43, 44]. The utility of the system is increased when the robot is used in conjunction with a soft-tissue tensioning device—the BalanceBot. So, unlike the Verasense sensor, The BalanceBot device is used in conjunction with the robot. It is inappropriate to include these two types of sensors together in our meta-analysis. There is also no article comparing OMNIBotics and BalanceBot device versus manual gap balance which could meet Inclusion criteria in our meta-analysis.

## Conclusion

Even though the use of intraoperative sensor technology was not related to an improvement in KSS, KSS Function score, OKS, KOOS, ROM, operative time, Mechanical axis, and rate of reoperation, the current studies showed that the ﻿Sensor use did result in a reduction in the rate of MUA. Given the relevant possible biases in our meta-analysis, more adequately powered and well-designed prospective studies with long-term follow-up were required to determine whether the application of the sensor technology for TKA will have clinical benefits and improve the survival of prostheses.

## Data Availability

The datasets generated during and/or analyzed during the current study are available from the corresponding author on reasonable request.

## Abbreviations

CIs: Confidence intervals
RCTs: Randomized controlled trials
RR: Risk ratio
OR: Odds ratio
VMD: Weighted mean difference
TKA: Total knee arthroplasty
BMI: Body Mass Index
KSS: Knee society score
KSFS: Knee Society Function Score
OKS: Oxford Knee Assessment
ROM: Range of motion
MUA: Manipulation under anesthesia
EMBASE: Excerpta medica database
CENTRAL: Cochrane Central Register of Controlled Trials
CNKI: China National Knowledge Infrastructure
RCT: Randomized controlled trial
